# Factors associated with survival of extremely preterm infants and development of a nomogram: a 9-year single-center study in China

**DOI:** 10.1038/s41598-026-55566-x

**Published:** 2026-05-28

**Authors:** Xia Lei, Wu Huijie, Lv Rongji, Sun Yanru, Du Wenjie, Zhao Jiawen, Shang Lihong, Dong Huifang

**Affiliations:** Department of Neonatology, The Third Affiliated Hospital of Zhengzhou University, Zhengzhou, China

**Keywords:** Extremely preterm infants, survival status, Associated factors, Nomogram, Prediction model, Diseases, Health care, Medical research, Risk factors

## Abstract

To investigate the clinical characteristics and factors associated with outcomes in extremely preterm infants with a gestational age < 28 weeks, and to develop a nomogram-based prediction model to support clinical decision-making in neonatal intensive care. We retrospectively collected clinical data from 722 extremely preterm infants admitted to a neonatal intensive care unit between January 2016 and December 2024. The cohort was randomly divided into a training set and a testing set at a ratio of 7:3. Univariate and multivariate logistic regression analyses were performed to identify independent factors associated with in-hospital mortality. A nomogram was constructed based on the multivariate model. The predictive performance of the model was evaluated using the receiver operating characteristic (ROC) curve, calibration curve, and decision curve analysis (DCA). This study included a total of 722 extremely preterm infants, of whom 390 survived to discharge and 332 died. Variables with statistical significance in univariate analyses were entered into multivariate logistic regression. Gestational age, a 1-minute Apgar score of 0–3, used of pulmonary surfactant, used of vasoactive drugs, and pulmonary hemorrhage were identified as independent factors associated with in-hospital mortality. The nomogram demonstrated excellent discrimination, with areas under the ROC curve (AUCs) of 0.969 and 0.953 in the training and testing sets, respectively. The sensitivity and specificity were 0.914 and 0.974 in the training set, and 0.838 and 0.966 in the testing set. The Hosmer–Lemeshow test indicated good calibration (χ² = 8.481, *P* = 0.388 in the training set; χ² = 10.011, *P* = 0.264 in the testing set). Decision curve analysis showed that the nomogram provided a favorable net benefit across a wide range of threshold probabilities. The nomogram developed in this study demonstrated good discrimination, calibration, and clinical utility for predicting mortality in extremely preterm infants. This model may serve as a practical tool for individualized risk assessment and early clinical intervention.

**Supplementary Information:**

The online version contains supplementary material available at 10.1038/s41598-026-55566-x.

## Introduction

Extremely preterm infants, defined as those born at a gestational age of less than 28 weeks, have profoundly immature organ systems and are highly susceptible to a wide range of complications. Consequently, they are associated with high rates of mortality and long-term morbidity. With advances in neonatal intensive care, the survival of extremely preterm infants has gradually improved over recent decades, and survival rates have shown a steady upward trend. Nevertheless, mortality among this population remains substantial and varies markedly across regions and clinical settings, with reported rates ranging from 10% to 90%^1^. Previous studies have shown that the average age at death is approximately 7 days, and 60–90% of deaths occur within the first 24 h after birth^2,3^. Therefore, identifying factors associated with mortality and developing reliable prediction tools remain important for improving outcomes in extremely preterm infants.

## Methods

### Study design and population

This study was designed as a retrospective cohort study. Extremely preterm infants admitted to the neonatal intensive care unit (NICU) between January 1, 2016 and December 31, 2024 were consecutively enrolled. Extremely preterm infants were defined as those born at a gestational age of less than 28 weeks.

The inclusion criteria were as follows: (1) gestational age < 28 weeks; and (2) admission to the NICU of our hospital after delivery(Patients transferred from other hospitals were excluded). The exclusion criteria were: (1) presence of severe congenital malformations or inherited metabolic diseases; (2) incomplete clinical data, including but not limited to missing information on birth weight, gestational age, Apgar score, major complications (e.g., sepsis, intraventricular hemorrhage, NRDS), or outcomes.

### Data collection

Clinical data of the infants and their mothers were retrospectively extracted from the electronic medical record system.

Infant-related variables included birth weight, gestational age, sex, Apgar scores at 1 and 5 min, singleton or multiple birth, and conception via assisted reproductive technology. Maternal variables included mode of delivery (vaginal delivery or cesarean section), gestational diabetes mellitus, hypertensive disorders of pregnancy, and abnormalities of amniotic fluid (polyhydramnios or oligohydramnios, meconium-stained amniotic fluid, or bloody amniotic fluid).

Treatment-related variables included antenatal corticosteroid exposure, postnatal use of pulmonary surfactant, and treatment for patent ductus arteriosus, including pharmacological or surgical intervention. Neonatal complications were also recorded, including neonatal respiratory distress syndrome (NRDS), intracranial hemorrhage, periventricular leukomalacia, and pulmonary hemorrhage.

### Definitions and diagnostic criteria

Gestational age was determined by obstetricians based on a comprehensive assessment of the last menstrual period and prenatal ultrasonography. The diagnoses of neonatal diseases, such as neonatal respiratory distress syndrome and pulmonary hemorrhage, were established according to the Practical Neonatology (5th edition). Maternal pregnancy-related complications, including hypertensive disorders of pregnancy and premature rupture of membranes, were diagnosed according to Obstetrics and Gynecology (10th edition).

### Outcome definitions and grouping

The primary outcome of this study was in-hospital mortality. Infants were categorized into three prognostic groups: (1)the survival group, defined as infants who were discharged after recovery or improvement or transferred to another hospital and confirmed to be alive during follow-up; (2) the in-hospital death group, defined as infants who died during hospitalization despite active treatment; and (3) the out-of-hospital death group, defined as infants whose parents abandoned treatment due to personal reasons and were discharged against medical advice, resulting in out-of-hospital death.

For further analysis of mortality, infants who died during hospitalization were divided into an early in-hospital death group (death occurring < 7 days after admission) and a late in-hospital death group (death occurring ≥ 7 days after admission).

### Model development and validation

Eligible infants were randomly assigned to a training set and a testing set at a ratio of 7:3 using computer-generated randomization. The training set was used to identify factors associated with mortality and to develop the prediction model, whereas the testing set was used to evaluate the performance and stability of the model.

A nomogram was constructed based on the results of multivariate logistic regression analysis. Internal validation was performed using the bootstrap resampling method with 1,000 repetitions.

### Statistical analysis

All statistical analyses were performed using R software (version 4.3.3). Continuous variables were assessed for normality and are presented as mean ± standard deviation or median (interquartile range), as appropriate. Categorical variables are expressed as numbers (percentages). Comparisons between groups were conducted using the Student’s t test、Mann-Whitney U test, chi-square test, or Fisher’s exact test, as appropriate.

Univariate logistic regression analysis was first performed to screen variables associated with in-hospital mortality. Variables with a P value < 0.05 in the univariate analysis were subsequently included in a multivariate logistic regression model to identify independent factors associated with in-hospital mortality.

The predictive performance of the nomogram was evaluated in terms of discrimination, calibration, and clinical utility. Discrimination was assessed using the receiver operating characteristic (ROC) curve and the area under the ROC curve (AUC). Calibration was evaluated using calibration curves and the Hosmer–Lemeshow goodness-of-fit test. Clinical utility was assessed using decision curve analysis (DCA). A two-sided P value < 0.05 was considered statistically significant.

A total of 722 extremely preterm infants were included, with 332 mortality events. The final model included 5 independent predictors, yielding an events-per-variable (EPV) of approximately 66, which substantially exceeds the recommended minimum of 10–20. The cohort was randomly split into training (70%) and testing (30%) sets, containing approximately 232 and 100 mortality events, respectively, ensuring adequate stability and reliability for model development and evaluation.

### Ethical approval

This study was approved by the Ethics Committee of the Thirstability and reliability for model development and to the retrospective nature of the study, the requirement for informed consent was waived by the ethics committee. All procedures were conducted in accordance with the Declaration of Helsinki.The authors declare no competing interests.Ethics approval number: 2023-05-15.

## Results

### Baseline characteristics and outcomes of extremely preterm infants

A total of 722 extremely preterm infants admitted to the neonatal intensive care unit (NICU) were included in this study. Among them, 390 infants survived to discharge, 80 died during hospitalization (51 early in-hospital deaths and 29 late in-hospital deaths), and 252 infants died after discharge following withdrawal of care. The overall mortality rate was 46.0%, including an in-hospital mortality rate of 11.1% and an out-of-hospital mortality rate of 34.9%.

**Fig. 1:**
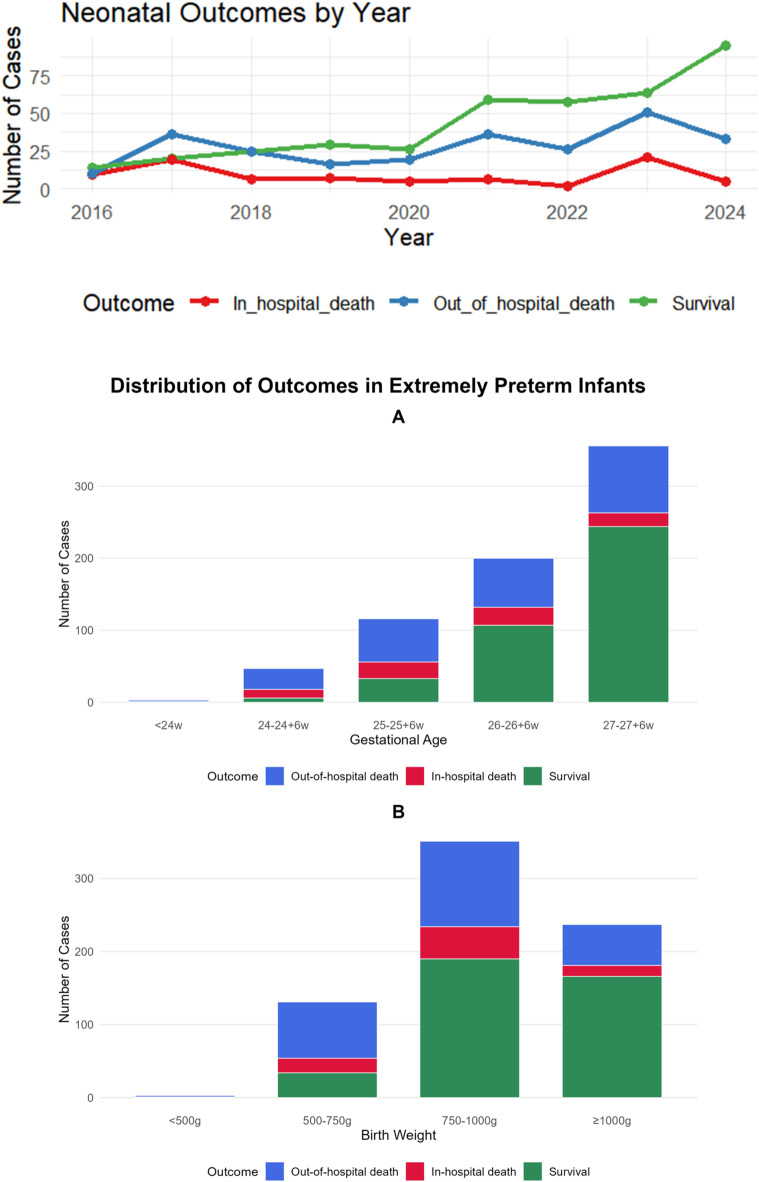
Trends in mortality by year, gestational age, and birth weight.

Mortality varied substantially according to gestational age and birth weight, with the highest mortality observed among infants born at < 25 weeks of gestation and those weighing < 750 g, reaching 80.0% and 74.6%, respectively. In-hospital mortality rates also differed across study years, peaking in 2017 at 73.3%. Detailed baseline characteristics and outcome distributions are presented Fig. 1.

### Comparison of clinical characteristics among outcome groups

Maternal characteristics, perinatal interventions, neonatal treatments, and clinical features were compared among infants with different outcomes (survival, early in-hospital death, late in-hospital death, and out-of-hospital death). Significant differences were observed across groups in several variables, including placental abruption, antenatal corticosteroid (ACS) administration (particularly completion of a full course), treatment for patent ductus arteriosus (PDA), use and repeated administration of pulmonary surfactant (PS), inhaled nitric oxide (NO) therapy, use of vasoactive drugs, gestational age, birth weight, multiple gestation, proportions of low Apgar scores at 1 and 5 min, cesarean delivery rate, and the incidence of severe complications such as grade 3–4 respiratory distress syndrome (RDS), grade 3–4 intraventricular hemorrhage (IVH), and pulmonary hemorrhage (all *P* < 0.05).

These findings suggest that perinatal management strategies, neonatal severity indicators, and major complications are closely associated with survival status and mortality patterns in extremely preterm infants. Detailed comparisons are shown in Table 1.

Gestational age and birth weight (non-normal, Shapiro-Wilk *P* < 0.05) are presented as median (IQR) and compared using Mann-Whitney U test. Survival group had higher gestational age than all death groups (*P* < 0.001) and higher birth weight than early in-hospital and out-of-hospital death groups (*P* < 0.001). SNK pairwise comparisons for categorical variables showed significant differences in: antenatal corticosteroids (early death group vs. others); adequate corticosteroids (early/late in-hospital death vs. survival/out-of-hospital death); PDA treatment (early in-hospital/out-of-hospital death vs. others); PS use (out-of-hospital death); vasoactive drugs and multiple-dose PS (survival group); severe RDS (early in-hospital death); pulmonary hemorrhage (late in-hospital death vs. survival/out-of-hospital death; early in-hospital death vs. all others).

**Table 1:** Univariate analysis of survival, in-hospital death, and Out-of-hospital death in extremely preterm infants.

	Survival (cases)	In-hospital death (cases)		Out-of-hospital Death Death (cases)	t/X²	*P*
		Early mortality	late mortality			
**Maternal pregnancy conditions**						
Placental Abruption	44(11.3)	10(19.6)	3(10.3)	53(21.0)	12.568	0.006
Premature Rupture of Membranes (PROM)≥18 h	163(41.8)	21(41.2)	11(37.9)	93(36.9)	1.608	0.657
Abnormal Amniotic Fluid	206(52.8)	20(39.2)	13(44.8)	126(50.0)	3.836	0.280
Gestational Hypertension	60(15.4)	7(13.7)	4(13.8)	48(19.0)	1.980	0.577
Gestational Diabetes Mellitus (GDM)	70(17.9)	3(5.9)	6(20.7)	31(12.3)	8.026	0.045
Cesarean Section	168(43.1)	12(23.5)	13(10.4)	66(26.2)	23.457	0.001
Genital Tract Infection	92(23.6)	11(21.6)	6(20.7)	62(24.6)	0.390	0.942
**Antenatal interventions**						
Cervical Cerclage	130(33.3)	14(27.5)	11(37.9)	62(24.6)	6.577	0.087
Antenatal Corticosteroids	344(88.2)	27(52.9)	24(82.8)	204(81.0)	40.827	0.001
Antenatal Corticosteroids Full Course of Treatment	174(44.6)	7(13.7)	6(20.7)	77(30.6)	29.441	0.001
Antenatal Magnesium Sulfate	220(56.4)	24(28.7)	17(58.6)	145(58.6)	1.991	0.574
**Postnatal treatments**						
Pulmonary Surfactant (PS) Administration	382(97.9)	49(96.1)	29(100)	218(86.5)	37.541	0.001
Multiple Doses of Pulmonary Surfactant (PS)	98(25.1)	33(64.7)	18(62.1)	86(34.1)	45.600	0.001
NO Administration	2(0.5)	4(7.8)	2(6.9)	18(7.1)	23.372	0.001
Vasoactive Drugs	105(26.9)	36(70.6)	24(82.8)	117(46.4)	74.447	0.001
**Characteristics of extremely preterm infants**						
Female	151(38.7)	14(27.5)	10(34.5)	109(43.3)	4.989	0.173
Gestational Age(w)	27.1(26.6,27.6)	26.0(25.3,26.7)	26.3(25.5,27.2)	26.4(25.6,27.3)	91.683	0.001
Birth Weight(g)	965.0(850.0, 1050.0)	850.0(715.0,950.0)	820.0(715.0,1010.0)	850.0(720.0,960.0)	73.872	0.001
Multiple Pregnancy	120(30.8)	27(52.9)	10(34.5)	105(41.7)	14.449	0.002
**Apgar scores**						
1-Minute Apgar Score (0-3points)	24(6.2)	10(19.6)	4(13.8)	49(19.4)	28.627	0.001
1-Minute Apgar Score (4-7points)	203(52.1)	35(68.6)	16(55.2)	142(56.3)	5.372	0.146
5-Minute Apgar Score (0-3points)	9(2.3)	3(5.9)	1(3.4)	10(4.0)	2.684	0.443
5-Minute Apgar Score (4-7points)	124(31.8)	27(52.9)	14(48.3)	129(51.2)	28.156	0.001
In Vitro Fertilization (IVF)	108(27.7)	12(23.5)	9(31.0)	58(23.0)	2.293	0.514
**Complications of extremely preterm infants**						
Respiratory Distress Syndrome (RDS) Grade 3–4	94(24.1)	27(52.9)	19(65.6)	86(34.1)	21.538	0.001
Intraventricular Hemorrhage (IVH) Grade 3–4	49(12.6)	8(15.7)	8(27.6)	78(31.0)	34.239	0.001
Pulmonary Hemorrhage	20(5.1)	26(51.0)	8(27.6)	37(14.7)	94.872	0.001
Cerebral Softening	13(3.3)	0(0.0)	1(3.4)	11(3.5)	2.463	0.461
Neonatal sepsis	131(33.6)	15(18.7)	14(17.5)	66(31.3)	4.922	0.085

### Univariate logistic regression analysis for extremely preterm infants mortality

Univariate logistic regression analysis was performed to compare the survival and mortality groups of extremely preterm infants. The results demonstrated clear differences in the distribution of several variables between the two groups, identifying multiple protective and risk factors significantly associated with adverse outcomes.

Higher gestational age (OR = 0.417, *P* < 0.001) and higher birth weight (OR = 0.996, *P* < 0.001) were significantly more common in the survival group and acted as protective factors. Cesarean delivery (OR = 0.570, *P* = 0.003) and antenatal corticosteroid exposure (OR = 0.411, *P* < 0.001), particularly completion of a full course (OR = 0.430, *P* < 0.001), were also observed at a higher frequency among survivors. Notably, the use of pulmonary surfactant (PS) was markedly more prevalent in the survival group and showed a strong protective effect (OR = 0.149, *P* < 0.001).

In contrast, multiple gestation (OR = 1.838, *P* < 0.001), severe placental abruption (OR = 1.639, *P* = 0.049), and signs of birth asphyxia—such as a 1-minute Apgar score of 0–3 (OR = 3.455, *P* < 0.001)—were significantly more frequent in the mortality group and were identified as definite risk factors.Regarding neonatal complications, severe respiratory distress syndrome (RDS grade 3–4; OR = 1.795, *P* = 0.003), repeated administration of PS (OR = 2.306, *P* < 0.001), use of vasoactive drugs (OR = 3.215, *P* < 0.001), pulmonary hemorrhage (OR = 6.649, *P* < 0.001), and intraventricular hemorrhage ≥ grade 3 (OR = 2.509, *P* < 0.001) occurred at significantly higher rates in the mortality group and were associated with an increased risk of adverse outcomes. Of particular concern, inhaled nitric oxide (NO) therapy (OR = 10.625, *P* = 0.002), as a marker of critical illness, showed the strongest association with mortality.

Sex, assisted reproductive technology, most maternal pregnancy complications, and antenatal infection did not differ significantly between the two groups in this univariate analysis. Detailed results are presented in Table 2.

**Table 2:** Univariate logistic regression analysis for extremely preterm infants overall mortality.

Variable	OR	CI lower	CI upper	*P* value
Gestational Age	0.417	0.334	0.515	0.001
Birth Weight	0.996	0.995	0.997	0.001
Female	1.085	0.756	1.556	0.657
Cesarean Section	0.570	0.390	0.827	0.003
Multiple Pregnancy	1.838	1.272	2.664	0.001
1-Minute Apgar Score (0-3points)	3.455	1.996	6.204	0.001
1-Minute Apgar Score (4-7points)	1.193	0.840	1.697	0.325
5-Minute Apgar Score (0-3points)	1.373	0.450	4.321	0.574
5-Minute Apgar Score (4-7points)	2.461	1.717	3.545	0.001
In Vitro Fertilization (IVF)	0.857	0.564	1.297	0.468
Gestational Hypertension	1.239	0.773	1.988	0.373
Gestational Diabetes Mellitus (GDM)	0.708	0.421	1.172	0.184
Premature Rupture of Membranes (PROM)≥18 h	0.790	0.550	1.133	0.202
Abnormal Amniotic Fluid	0.789	0.555	1.120	0.185
Placental Abruption	1.639	1.005	2.696	0.049
Cervical Cerclage	0.733	0.498	1.076	0.114
Genital Tract Infection	0.836	0.549	1.266	0.400
Antenatal Magnesium Sulfate	0.970	0.683	1.378	0.864
Antenatal Corticosteroids	0.411	0.250	0.664	0.001
Antenatal Corticosteroids Full Course of Treatment	0.430	0.292	0.626	0.001
Respiratory Distress Syndrome (RDS) Grade 3–4	1.795	1.222	2.648	0.003
Pulmonary Surfactant (PS) Administration	0.149	0.050	0.364	0.001
Multiple Doses of Pulmonary Surfactant (PS)	2.306	1.581	3.382	0.001
NO Administration	10.625	3.003	67.481	0.002
Vasoactive Drugs	3.215	2.222	4.684	0.001
Pulmonary Hemorrhage	6.649	3.499	13.763	0.001
Intraventricular Hemorrhage (IVH) Grade 3–4	2.509	1.597	3.998	0.001
Cerebral Softening	1.326	0.499	3.586	0.568
neonatal sepsis	0.233	0.919	1.733	0.151

### Multivariable logistic regression analysis of mortality in extremely preterm infants

After adjustment for potential confounders, multivariate logistic regression analysis identified several independent predictors of mortality. Higher gestational age (adjusted OR [aOR] = 0.655, 95% CI: 0.478–0.891, *P* = 0.008) and pulmonary surfactant administration (aOR = 0.113, 95% CI: 0.035–0.309, *P* = 0.001) remained significant protective factors. Conversely, severe perinatal asphyxia (Apgar score 0–3 at 1 min; aOR = 2.061, 95% CI: 1.040–4.175, *P* = 0.041), use of vasoactive drugs (aOR = 1.972, 95% CI: 1.224–3.178, *P* = 0.005), and pulmonary hemorrhage (aOR = 4.790, 95% CI: 2.318–10.648, *P* = 0.001) were identified as independent risk factors for mortality.

**Fig. 2:**
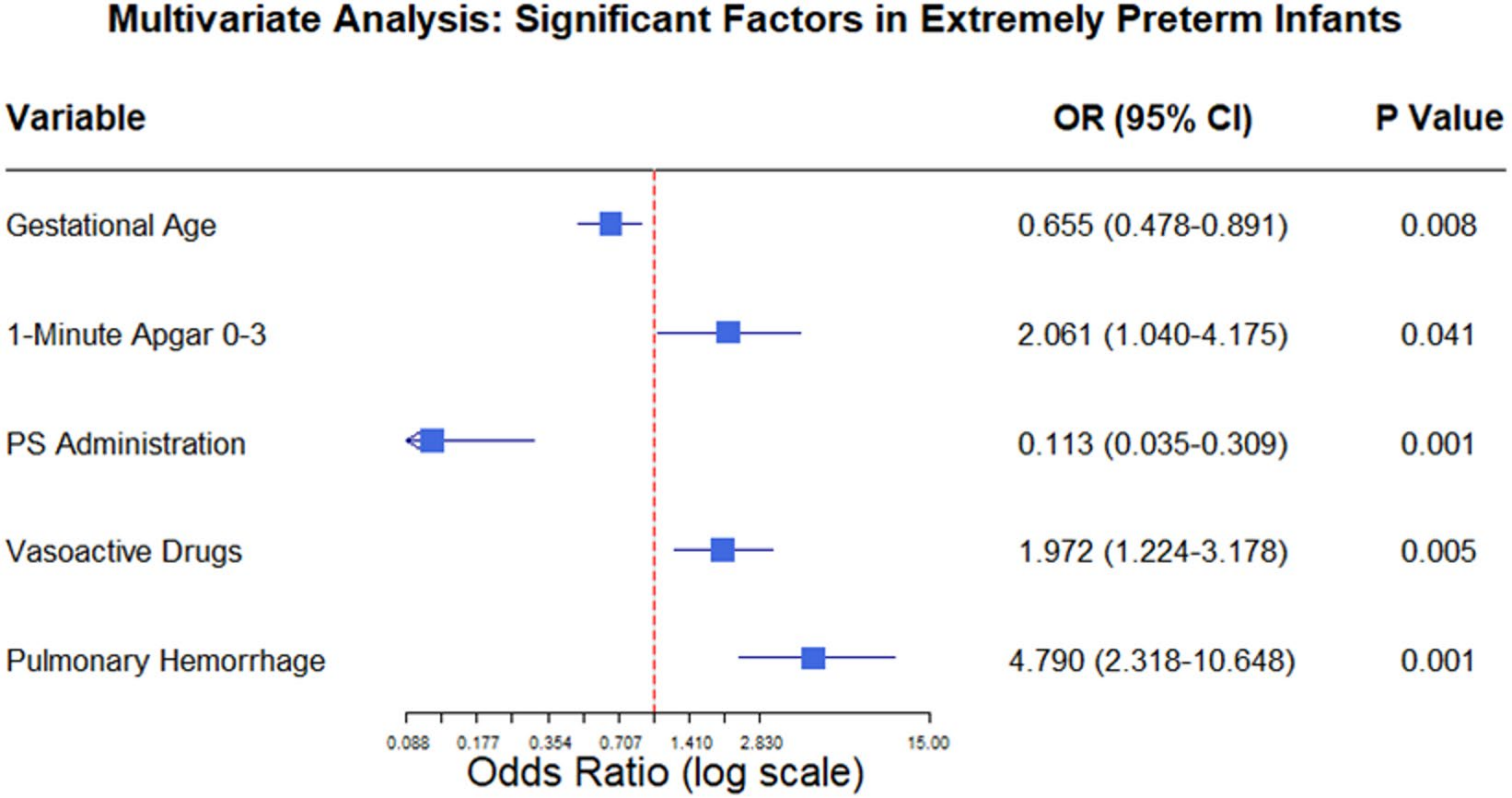
Forest plot showing multivariable logistic regression results for mortality in extremely preterm infants.

Cesarean delivery (aOR = 0.607, 95% CI: 0.364–1.003, *P* = 0.053) and repeated surfactant administration (aOR = 1.604, 95% CI: 0.973–2.641, *P* = 0.063) showed borderline statistical significance. Other variables, including birth weight, multiple gestation, antenatal corticosteroid exposure, and severe IVH, did not retain independent predictive value after adjustment. The multivariate results are summarized in Table 3, with a forest plot shown in Fig. 2.

**Table 3:** Multivariable logistic regression analysis of overall mortality in extremely preterm infants.

Variable	OR	CI lower	CI upper	*P* value
Gestational Age	0.655	0.478	0.891	0.008
Birth Weight	0.999	0.997	1.000	0.168
1-Minute Apgar Score (0-3points)	2.061	1.040	4.175	0.041
5-Minute Apgar Score (4-7points)	1.337	0.842	2.116	0.216
Antenatal Corticosteroids	0.706	0.381	1.303	0.266
Antenatal Corticosteroids Full Course of Treatment	0.654	0.406	1.049	0.079
Pulmonary Surfactant(PS)Administration	0.113	0.035	0.309	0.001
Multiple Doses of Pulmonary Surfactant(PS)	1.604	0.973	2.641	0.063
Vasoactive Drugs	1.972	1.224	3.178	0.005
Pulmonary Hemorrhage	4.790	2.318	10.648	0.001
Intraventricular Hemorrhage (IVH) Grade 3–4	1.366	0.782	2.387	0.272
Multiple Pregnancy	0.982	0.606	1.581	0.941
NO Administration	3.461	0.800	24.409	0.137
Cesarean Section	0.607	0.364	1.003	0.053
Respiratory Distress Syndrome (RDS) Grade 3–4	1.421	0.861	2.344	0.168
Placental Abruption	1.324	0.716	2.446	0.369

**Fig. 3:**
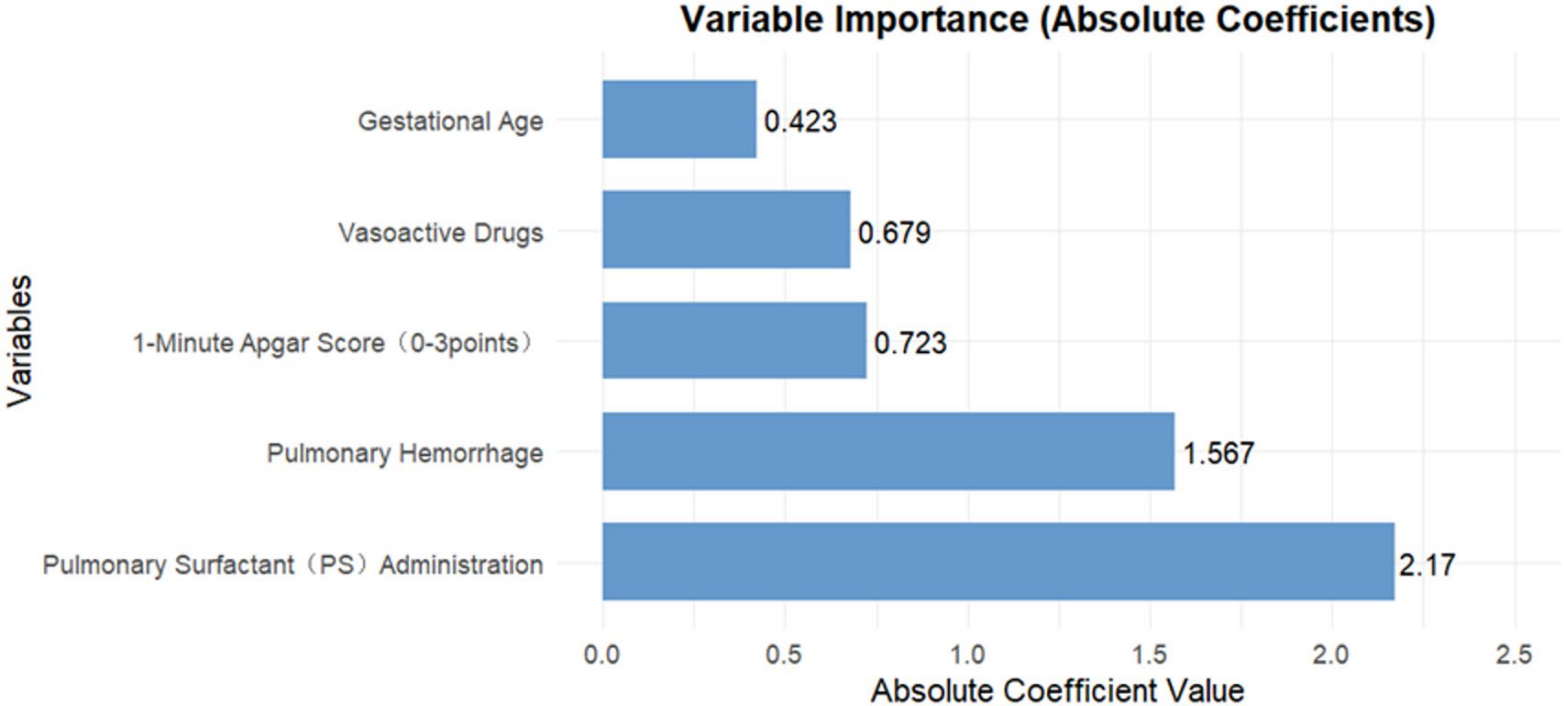
Variable importance for mortality in extremely preterm infants based on multivariable logistic regression.

 Variable importance analysis further highlighted pulmonary surfactant administration as the most influential predictor of outcome, demonstrating both strong protective effects and the highest relative importance score (importance = 2.17). The variable importance ranking is presented in Fig. 3.

### Nomogram for predicting in-hospital mortality in extremely preterm infants

**Fig. 4:**
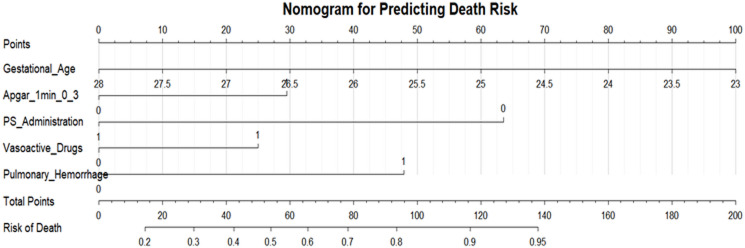
Nomogram for predicting in-hospital mortality in extremely preterm infants.

Based on the multivariate logistic regression model derived from the training cohort, five independent predictors were incorporated to construct a nomogram for individualized risk prediction. The nomogram allows clinicians to assign points to each predictor according to the infant’s clinical characteristics, sum the total score, and estimate the probability of in-hospital mortality during the observation period. The graphical representation of the nomogram is shown in Fig. 4.

### Model performance and clinical utility of the nomogram for in-hospital mortality in extremely preterm infants

**Fig. 5:**
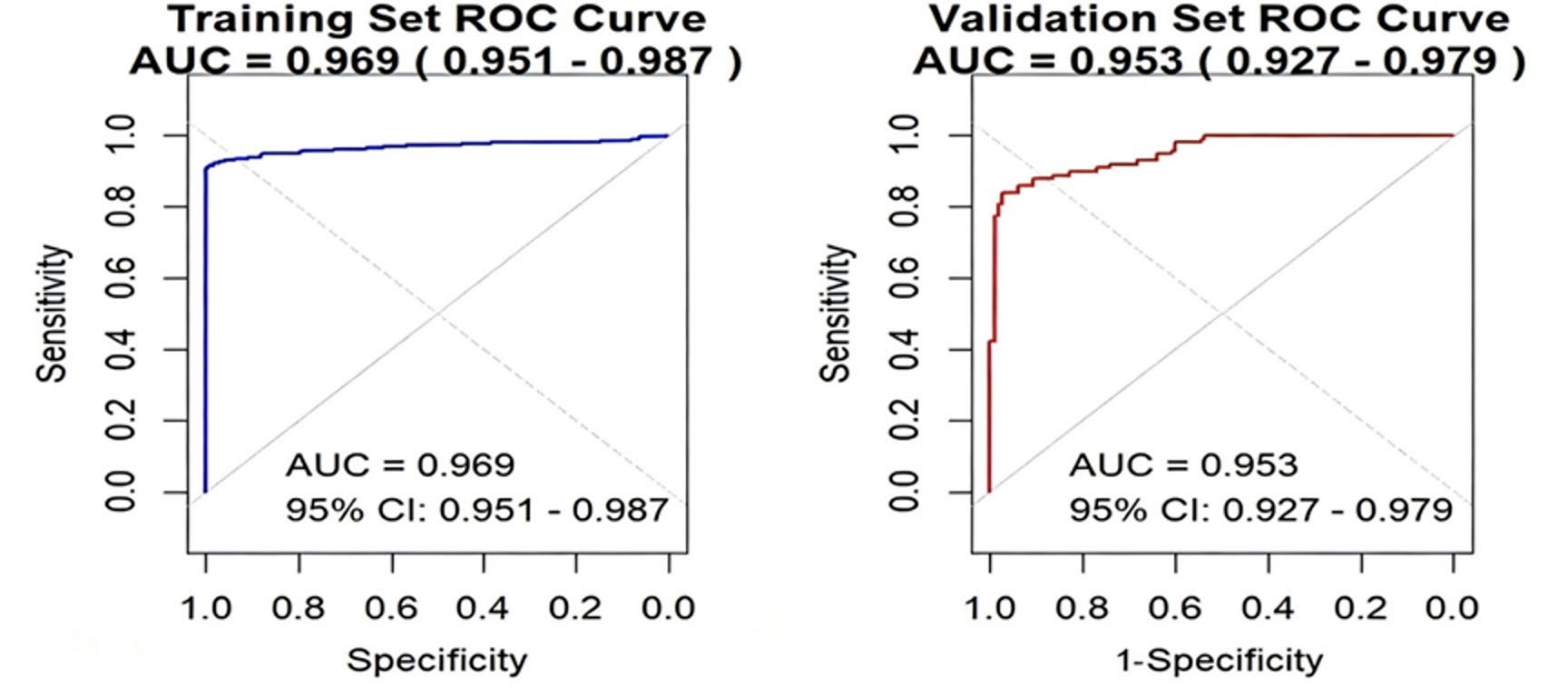
Receiver operating characteristic (ROC) curves of the nomogram for predicting mortality in extremely preterm infants.

The discriminatory performance of the nomogram was assessed using receiver operating characteristic (ROC) curves. In the training cohort, the area under the curve (AUC) was 0.969 (95% CI: 0.951–0.987), with a sensitivity of 0.914 and a specificity of 0.966. In the testing cohort, the model also demonstrated excellent discrimination, with an AUC of 0.953 (95% CI: 0.927–0.979), sensitivity of 0.838, and specificity of 0.974. ROC curves are shown in Fig. 5.

**Fig. 6:**
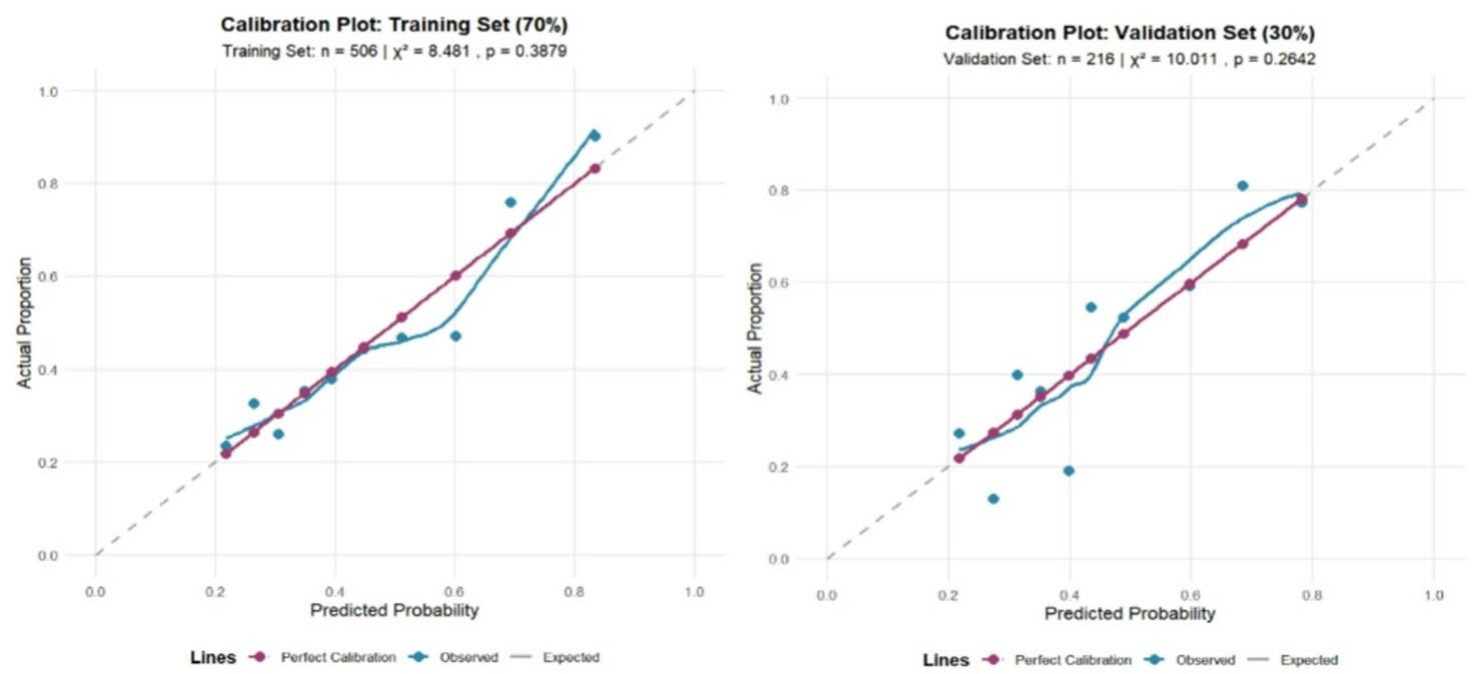
Calibration curves of the nomogram in the training and validation cohorts.

 Calibration of the nomogram was evaluated using bootstrap resampling (1,000 iterations). Calibration curves for both the training and testing cohorts demonstrated good agreement between predicted and observed outcomes. The Hosmer–Lemeshow test yielded χ² values of 8.481 (*P* = 0.388) and 10.011 (*P* = 0.264) for the training and testing cohorts, respectively, indicating satisfactory calibration. Calibration plots are presented in Fig. 6.

**Fig. 7:**
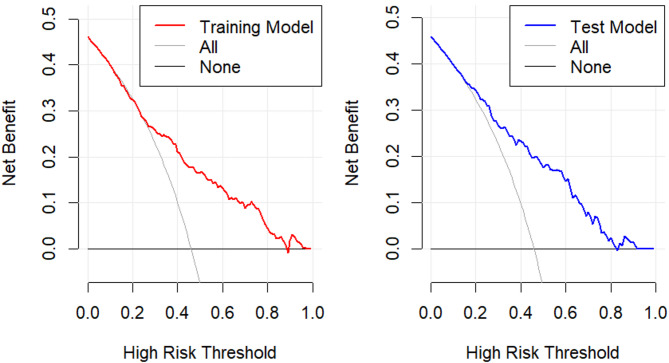
Decision curve analysis of the nomogram for predicting mortality in extremely preterm infants.

Clinical utility was assessed using decision curve analysis (DCA). The nomogram provided a positive net benefit across a wide range of threshold probabilities, specifically between 20% and 99% in the training cohort and 10% to 99% in the testing cohort, supporting its potential clinical applicability in guiding decision-making for extremely preterm infants. The DCA results are shown in Fig. 7.

## Discussion

In conclusion, this study developed and internally validated a nomogram-based prediction model for in-hospital mortality among extremely preterm infants using a large, real-world cohort from a tertiary perinatal center in China. The model integrates five readily obtainable clinical variables and demonstrated excellent discrimination, good calibration, and consistent clinical utility. By enabling individualized risk stratification early in hospitalization, this tool may support clinical decision-making, optimize resource allocation, and facilitate transparent communication with families. Further multicenter prospective studies with external validation are warranted to confirm its generalizability and to explore its integration into routine neonatal care.

Extremely preterm infants, defined as those born at a gestational age of less than 28 weeks, are at exceptionally high risk of mortality due to profound physiological immaturity and a high burden of complications. A 10-year report from a perinatal center in Shandong, China, published in 2022, documented a mortality rate of 79.3% among extremely preterm infants^4^, while studies from neonatal networks in developed countries have reported mortality rates ranging from 45.7% to 84% ^5–8^. In the present study, the overall mortality rate was 46.0%, which is broadly consistent with these previous reports. The survival of extremely preterm infants is widely regarded as an indicator of the comprehensive capacity of a perinatal center. In recent years, increasing efforts have been devoted to developing predictive models for various preterm-related outcomes, including length of hospital stay^9^, timing of surgical intervention for necrotizing enterocolitis^10^, and bronchopulmonary dysplasia^11^. Several centers in China have also explored prognostic models for extremely preterm infants^12;]^ however, large-sample tools specifically designed to predict survival outcomes remain limited. To address this gap, we developed and validated a nomogram-based model using 9 years of clinical data from a tertiary perinatal center in China. The model incorporates five readily available clinical variables—gestational age, 1-minute Apgar score (0–3), pulmonary surfactant administration, use of vasoactive drugs, and pulmonary hemorrhage—and demonstrated excellent discrimination (AUC > 0.95), good calibration, and broad clinical net benefit in both training and testing cohorts, supporting its stability, reliability, and practical clinical value.

Gestational age remains the most critical determinant of survival in extremely preterm infants. As gestational age decreases, immaturity of multiple organ systems becomes more pronounced, physiological reserves are limited, and the ability to adapt to extrauterine life is substantially compromised, resulting in a markedly increased risk of death. Previous studies have reported survival rates of 50%–91.4% for infants born at 24–26 weeks’ gestation in developed countries^13,14^, whereas data from the Chinese Neonatal Network indicate survival rates ranging from 21.7% to 89.0% for infants born at 23–27 weeks^15^. Consistent with these findings, our study demonstrated that infants born at ≥ 27 weeks had nearly twice the survival rate of those born at < 26 weeks (68.5% vs. 23.5%). Although the overall survival rate in our center was lower than that reported in some previous studies—potentially reflecting regional economic disparities and differences in healthcare resources—a clear upward trend was observed over time, with survival increasing from 42.4% to 71.4%, suggesting a gradual improvement in the quality of care for extremely preterm infants at our institution.

A low Apgar score is a simple and widely used indicator of perinatal asphyxia and an important predictor of neonatal adverse outcome Previous research has shown that neonates with Apgar scores of 0 or 1 have a fivefold higher risk of death compared with those scoring 9 or 10^16^. Low Apgar scores have also been strongly associated with adverse neurological outcomes, including neurodevelopmental impairment^17^ and cerebral palsy^18^. A Malaysian study involving 1,580 neonates with a 1-minute Apgar score < 4 reported that only 29.4% recovered to a 5-minute Apgar score ≥ 7^19,]^ highlighting the difficulty of rapid physiological recovery among infants with severe birth asphyxia. These findings underscore the importance of prompt, effective, and sustained resuscitation and intensive monitoring for extremely preterm infants presenting with severe asphyxia at birth.

Respiratory complications are highly prevalent among extremely preterm infants due to profound pulmonary immaturity^20^. Insufficient endogenous pulmonary surfactant production is the central mechanism underlying the high incidence of neonatal respiratory distress syndrome (NRDS), which affects approximately 80% of this population^21,22^. Exogenous surfactant replacement therapy effectively reduces alveolar surface tension and prevents alveolar collapse and has become the cornerstone of NRDS management^23^. Our findings further confirm the critical role of surfactant therapy, demonstrating a strong and independent protective association with mortality after multivariate adjustment. Pulmonary hemorrhage, one of the most severe complications of NRDS, is associated with a substantial increase in mortality and worsened long-term outcomes^24^. Previous studies have reported that extremely preterm infants account for 42%–45% of neonatal pulmonary hemorrhage cases^25^. In our cohort, the mortality rate among extremely preterm infants with pulmonary hemorrhage reached 78%. These infants also exhibited a higher incidence of shock, which may further exacerbate lung injury and adversely affect prognosis. A multicenter study from the United States reported that pulmonary hemorrhage occurs most frequently in infants born at 23–24 weeks’ gestation, with most cases developing within the first 7 days after birth, and that the risk decreases with increasing gestational age^26^. The use of vasoactive and inotropic agents—such as dopamine, dobutamine, epinephrine, milrinone, vasopressin, and norepinephrine—reflects hemodynamic instability and severe illness^27,28^. When circulatory failure progresses to the point that such agents are required to maintain life, the risk of mortality increases substantially.

A notable finding was that out-of-hospital deaths following treatment withdrawal (252 cases) far exceeded in-hospital deaths (80 cases). This reflects real-world challenges in managing extremely preterm infants in China, where decisions are influenced by parental preferences, socioeconomic burden, and concerns about neurodevelopmental outcomes. While a high proportion of out-of-hospital deaths is also common in low- and middle-income countries, China’s unique context makes this issue even more complex.^29^. In addition to economic burden, traditional beliefs regarding childbearing, societal acceptance of children with disabilities, and the lack of comprehensive long-term care and support systems are all important factors influencing family decision-making. Moreover, end-of-life decisions for critically ill infants may vary substantially across different countries, cultures, and ethnic backgrounds^30–34^. In addition, limited capacity for the care of extremely preterm infants in some primary or lower-level medical institutions, as well as the risks associated with inter-hospital transport, may further contribute to parental uncertainty regarding the prospects of intensive treatment^35^.Post-hoc comparisons in Table 1 revealed that the out-of-hospital death group had better rates of adequate antenatal corticosteroid (ACS) administration and PDA treatment than the in-hospital death group, but lower rates than the survival group, suggesting that some out-of-hospital deaths might have been salvageable. Therefore, our prediction model carries important social implications: an objective and quantitative risk assessment tool can facilitate more intuitive and effective communication between clinicians and families, help parents make informed and rational decisions, and potentially reduce unnecessary treatment abandonment caused by information asymmetry or excessive anxiety.

In summary, this study developed and validated a high-performance, easy-to-use clinical prediction model for mortality risk in extremely preterm infants. The model confirmed the central roles of gestational age, asphyxia, severe complications, and hemodynamic instability in prognostic assessment, and transformed multiple risk factors into an intuitive, individualized risk probability, facilitating early risk stratification. However, this study has limitations, including selection bias inherent to its single-center retrospective design and the potential impact of evolving clinical practices on model timeliness. Moreover, non-clinical variables such as parental willingness and family socioeconomic status were not included. Future multicenter prospective studies are needed for external validation, and efforts should be made to integrate the model with indicators of family support systems to establish a bio-social integrated prediction system.

## Supplementary Information

Below is the link to the electronic supplementary material.

Supplementary Material 1

Supplementary Material 2

## Data Availability

The data that support the findings of this study are available from the Third Affiliated Hospital of Zhengzhou University but restrictions apply to the availability of these data, which were used under license for the current study. Data are therefore not publicly available but may be available from the corresponding author upon reasonable request and with permission of the Third Affiliated Hospital of Zhengzhou University.

## References

[1] Blencowe, H. et al. Born too soon: the global epidemiology of 15 million preterm births. *Reprod. Health*. **10** (Suppl 1). 10.1186/1742-4755-10-S1-S2 (2013). 10.1186/1742-4755-10-S1-S2 PMC3828585 24625129

[2] Puia-Dumitrescu, M. et al. Medications and in-hospital outcomes in infants born at 22–24 weeks of gestation. *J. Perinatol.* **40**, 781–789. 10.1038/s41372-020-0614-4 (2020). 32066843 10.1038/s41372-020-0614-4 PMC7293630

[3] Mehler, K. et al. Survival among infants born at 22 or 23 weeks’ gestation following active prenatal and postnatal care. *JAMA Pediatr.* **170**, 671–677. 10.1001/jamapediatrics.2016.0207 (2016). 27214875 10.1001/jamapediatrics.2016.0207

[4] Perlbarg, J. et al. Delivery room management of extremely preterm infants: the EPIPAGE-2 study. *Arch. Dis. Child. Fetal Neonatal Ed.* **101**, F384–F390 (2016). 26837310 10.1136/archdischild-2015-308728

[5] Ishii, N., Kono, Y., Yonemoto, N., Kusuda, S. & Fujimura, M. Outcomes of infants born at 22 and 23 weeks’ gestation. *Pediatrics* **132**, 62–71 (2013). 23733804 10.1542/peds.2012-2857

[6] Vilanova, C. S. et al. The relationship between low birth weight strata and infant mortality in southern Brazil. *Popul. Health Metr.* **17**, 15 (2019). 31775758 10.1186/s12963-019-0195-7 PMC6882357

[7] Owens, J. D. et al. Multi-hospital community NICU quality improvement improves survival of ELBW infants. *J. Miss. State Med. Assoc.* **56**, 237–242 (2015). 26521537

[8] Zhang, W. W., Yu, Y. H., Dong, X. Y. & Reddy, S. Treatment status of extremely premature infants with gestational age < 28 weeks in a Chinese perinatal center from 2010 to 2019. *World J. Pediatr.* **18**, 67–74 (2022). 34767193 10.1007/s12519-021-00481-6 PMC8761149

[9] Yang, Y. et al. Construction and validation of a risk prediction model for prolonged hospitalization of very premature infants. *Value Health*. **28**, 1565–1573 (2025). 40614819 10.1016/j.jval.2025.06.011

[10] Nayak, S. P. et al. Development of a prediction model for surgery or early mortality at initial assessment for necrotizing enterocolitis. *Am. J. Perinatol.* **41**, 1714–1727 (2024). 38272063 10.1055/a-2253-8656

[11] Kostekci, Y. E. et al. An early prediction model for estimating bronchopulmonary dysplasia in preterm infants. *Neonatology* **120**, 709–717 (2023). 37725910 10.1159/000533299 PMC10711771

[12] Xu, J. et al. Establishment of a nomogram prediction model for early death risk in extremely preterm infants. *Chin. J. Perinat. Med.* **27**, 394–401. 10.3760/cma.j.cn113903-20230828-00163 (2024).

[13] van Beek, P. E. et al. Survival and causes of death in extremely preterm infants in the Netherlands. *Arch. Dis. Child. Fetal Neonatal Ed.* **106**, 251–257. 10.1136/archdischild-2020-318978 (2021). 33158971 10.1136/archdischild-2020-318978 PMC8070636

[14] Schindler, T. et al. Causes of death in very preterm infants cared for in neonatal intensive care units. *BMC Pediatr.* **17**, 59. 10.1186/s12887-017-0810-3 (2017). 28222717 10.1186/s12887-017-0810-3 PMC5319155

[15] Cao, Y. et al. Assessment of NICU practices, morbidity, and mortality among very preterm infants in China. *JAMA Netw. Open.* **4**, e2118904. 10.1001/jamanetworkopen.2021.18904 (2021). 34338792 10.1001/jamanetworkopen.2021.18904 PMC8329742

[16] Cnattingius, S., Johansson, S. & Razaz, N. Apgar score and risk of neonatal death among preterm infants. *N Engl. J. Med.* **383**, 49–57 (2020). 32609981 10.1056/NEJMoa1915075

[17] Amar, W. & Eyal, S. Low five-minute Apgar score and neurological morbidities: does prematurity modify the association? *J. Clin. Med.* **11**, 1922 (2022). 35407530 10.3390/jcm11071922 PMC8999413

[18] Osredkar, D. et al. Apgar score and risk of cerebral palsy in preterm infants. *Neuropediatrics* **52**, 310–315 (2021). 34162009 10.1055/s-0041-1729181

[19] Jeganathan, R. et al. Factors associated with recovery from 1-minute Apgar score < 4. *BMC Pregnancy Childbirth*. **17**, 110 (2017). 28390414 10.1186/s12884-017-1293-9 PMC5385027

[20] Abiramalatha, T. et al. Interventions to prevent bronchopulmonary dysplasia in preterm neonates: an umbrella review. *JAMA Pediatr.* **176**, 502–516 (2022). 35226067 10.1001/jamapediatrics.2021.6619

[21] De Luca, D. Respiratory distress syndrome in preterm neonates in the era of precision medicine. *Pediatr. Neonatol*. **62** (Suppl 1), 3–S9. 10.1016/j.pedneo.2020.11.005 (2021). 33358440 10.1016/j.pedneo.2020.11.005

[22] Boussuges, A. et al. Diagnosis of hemidiaphragm paralysis: refined ultrasound criteria. *Front. Med.* **11**, 1416520. 10.3389/fmed.2024.1416520 (2024). 10.3389/fmed.2024.1416520 PMC11153810 38846144

[23] Possmayer, F., Zuo, Y. Y., Veldhuizen, R. A. W. & Petersen, N. O. Pulmonary surfactant: a mighty thin film. *Chem. Rev.* **123**, 13209–13290. 10.1021/acs.chemrev.3c00146 (2023). 37862151 10.1021/acs.chemrev.3c00146

[24] Lee, M. et al. Pulmonary hemorrhage in neonatal respiratory distress syndrome. *J. Neonatal Perinat. Med.* **12**, 161–171 (2019). 10.3233/NPM-1867 31256080

[25] Clyman, R. I. & Hills, N. K. Prophylactic indomethacin and risk of pulmonary hemorrhage in infants < 28 weeks. *J. Perinatol.* **44**, 1470–1477 (2024). 38658692 10.1038/s41372-024-01971-x

[26] Ahmad, K. A. et al. Morbidity and mortality with early pulmon*ary haemor*rhage in preterm neonates. *Arch. Dis. Child. Fetal Neonatal Ed.* **104**, F63–F68. 10.1136/archdischild-2017-314172 (2019). 29374627 10.1136/archdischild-2017-314172

[27] Haque, A. et al. Association between vasoactive-inotropic score and mortality in pediatric septic shock. *Indian Pediatr.* **52**, 311–313 (2015). 25929629 10.1007/s13312-015-0630-1

[28] Crow, S. S. et al. Duration and magnitude of vasopressor support predicts poor outcome after infant cardiac operations. *Ann. Thorac. Surg.* **98**, 655–661. 10.1016/j.athoracsur.2014.04.041 (2014). 24906599 10.1016/j.athoracsur.2014.04.041

[29] Blencowe, H. et al. National, regional, and worldwide estimates of preterm birth rates in the year 2010 with time trends since 1990 for selected countries: a systematic analysis and implications. *Lancet* **379**, 2162–2172. 10.1016/S0140-6736(12)60820-4 (2012). 22682464 10.1016/S0140-6736(12)60820-4

[30] Zhong, Y., Cavolo, A., Labarque, V. & Gastmans, C. Physician decision-making process about withholding or withdrawing life-sustaining treatments in paediatric patients: a systematic review of qualitative evidence. *BMC Palliat. Care*. **21**, 113. 10.1186/s12904-022-01003-5 (2022). 35751075 10.1186/s12904-022-01003-5 PMC9229823

[31] Fu, K. X., Chiong, Y. K. & Ngiam, N. End-of-life decision making in critical illness: perspectives of Asian parents. *Palliat. Support Care*. **20**, 233–242. 10.1017/S1478951521000493 (2022). 33942708 10.1017/S1478951521000493

[32] Chan, L. C. et al. End-of-life decision-making for newborns: a 12-year experience in Hong Kong. *Arch. Dis. Child. Fetal Neonatal Ed.* **101**, F37–F42. 10.1136/archdischild-2015-308659 (2016). 26271752 10.1136/archdischild-2015-308659

[33] Aladangady, N. & de Rooy, L. Withholding or withdrawal of life-sustaining treatment for newborn infants. *Early Hum. Dev.* **88**, 65–69. 10.1016/j.earlhumdev.2012.01.002 (2012). 22261290 10.1016/j.earlhumdev.2012.01.002

[34] Michelson, K. N. et al. Parental views on withdrawing life-sustaining therapies in critically ill children. *Arch. Pediatr. Adolesc. Med.* **163**, 986–992. 10.1001/archpediatrics.2009.180 (2009). 19884588 10.1001/archpediatrics.2009.180 PMC2873853

[35] Mohamed, M. A. & Aly, H. Transport of premature infants is associated with increased risk for intraventricular haemorrhage. *Arch. Dis. Child. Fetal Neonatal Ed.* **95**, F403–F407. 10.1136/adc.2010.183236 (2010). 20584801 10.1136/adc.2010.183236

