# Supplementary material for: Factors associated with survival of extremely preterm infants and development of a nomogram: a 9-year single-center study in China
Source: Sci Rep. 2026 May 28;16:24480. doi: 10.1038/s41598-026-55566-x (PMC13451414; doi:10.1038/s41598-026-55566-x)
Supplement: Supplementary file 1 [file 41598_2026_55566_MOESM1_ESM.docx]

****Figure 1 . Trends in mortality by year, gestational age, and birth weight****

****Figure 2 Forest plot showing multivariable logistic regression results for mortality in extremely preterm infants****

****Figure 3 . Variable importance for mortality in extremely preterm infants based on multivariable logistic regression****

****Figure 4.Nomogram for predicting in-hospital mortality in extremely preterm infants****

****Figure 5. Receiver operating characteristic (ROC) curves of the nomogram for predicting mortality in extremely preterm infants****

****Figure 6. Calibration curves of the nomogram in the training and validation cohorts****

****Figure 7. Decision curve analysis of the nomogram for predicting mortality in extremely preterm infants****
