# Supplementary material for: Factors associated with survival of extremely preterm infants and development of a nomogram: a 9-year single-center study in China
Source: Sci Rep. 2026 May 28;16:24480. doi: 10.1038/s41598-026-55566-x (PMC13451414; doi:10.1038/s41598-026-55566-x)
Supplement: Supplementary file 2 [file 41598_2026_55566_MOESM2_ESM.docx]

****Table 1.Univariate analysis of survival, in-hospital death, and Out-of-hospital death in extremely preterm infants****

|  | ****Survival****  ****(cases)**** | ****In-hospital death (cases)**** | | **Out-of-hospital **Death Death****  ****(cases)**** | ****t/X²**** | ****P**** |
| --- | --- | --- | --- | --- | --- | --- |
|  |  | ****Early mortality**** | ****late mortality**** |  |  |  |
| ****Maternal pregnancy conditions**** |  |  |  |  |  |  |
| **Placental Abruption** | **44（11.3）** | **10（19.6）** | **3（10.3）** | **53（21.0）** | **12.568** | **0.006** |
| **Premature Rupture of Membranes (PROM)≥18h** | **163（41.8）** | **21（41.2）** | **11（37.9）** | **93（36.9）** | **1.608** | **0.657** |
| **Abnormal Amniotic Fluid** | **206（52.8）** | **20（39.2）** | **13（44.8）** | **126（50.0）** | **3.836** | **0.280** |
| **Gestational Hypertension** | **60（15.4）** | **7（13.7）** | **4（13.8）** | **48（19.0）** | **1.980** | **0.577** |
| **Gestational Diabetes Mellitus (GDM)** | **70(17.9)** | **3(5.9)** | **6(20.7)** | **31(12.3)** | **8.026** | **0.045** |
| **Cesarean Section** | **168（43.1）** | **12（23.5）** | **13（10.4）** | **66（26.2）** | **23.457** | **0.001** |
| **Genital Tract Infection** | **92（23.6）** | **11（21.6）** | **6（20.7）** | **62（24.6）** | **0.390** | **0.942** |
| ****Antenatal interventions**** |  |  |  |  |  |  |
| **Cervical Cerclage** | **130（33.3）** | **14（27.5）** | **11（37.9）** | **62（24.6）** | **6.577** | **0.087** |
| **Antenatal Corticosteroids** | **344（88.2）** | **27（52.9）** | **24（82.8）** | **204（81.0）** | **40.827** | **0.001** |
| **Antenatal Corticosteroids Full Course of Treatment** | **174（44.6）** | **7（13.7）** | **6（20.7）** | **77（30.6）** | **29.441** | **0.001** |
| **Antenatal Magnesium Sulfate** | **220（56.4）** | **24（28.7）** | **17（58.6）** | **145（58.6）** | **1.991** | **0.574** |
| ****Postnatal treatments**** |  |  |  |  |  |  |
| **Pulmonary Surfactant (PS) Administration** | **382（97.9)** | **49(96.1)** | **29(100)** | **218(86.5)** | **37.541** | **0.001** |
| **Multiple Doses of Pulmonary Surfactant (PS)** | **98(25.1)** | **33(64.7)** | **18(62.1)** | **86(34.1)** | **45.600** | **0.001** |
| **NO Administration** | **2(0.5)** | **4(7.8)** | **2(6.9)** | **18(7.1)** | **23.372** | **0.001** |
| **Vasoactive Drugs** | **105(26.9)** | **36(70.6)** | **24(82.8)** | **117(46.4)** | **74.447** | **0.001** |
| ****Characteristics of extremely preterm infants**** |  |  |  |  |  |  |
| **Female** | **151（38.7）** | **14（27.5）** | **10（34.5）** | **109（43.3）** | **4.989** | **0.173** |
| **Gestational Age（w）** | **27.1（26.6,27.6）** | **26.0（25.3,26.7）** | **26.3（25.5,27.2）** | **26.4（25.6,27.3）** | **91.683** | **0.001** |
| **Birth Weight（g）** | **965.0（850.0，1050.0）** | **850.0（715.0,950.0）** | **820.0（715.0,1010.0）** | **850.0（720.0,960.0）** | **73.872** | **0.001** |
| **Multiple Pregnancy** | **120（30.8）** | **27(52.9)** | **10(34.5)** | **105(41.7)** | **14.449** | **0.002** |
| ****Apgar scores**** |  |  |  |  |  |  |
| **1-Minute Apgar Score (0-3points)** | **24(6.2)** | **10(19.6)** | **4(13.8)** | **49(19.4)** | **28.627** | **0.001** |
| **1-Minute Apgar Score (4-7points)** | **203（52.1）** | **35（68.6）** | **16（55.2）** | **142（56.3）** | **5.372** | **0.146** |
| **5-Minute Apgar Score (0-3points)** | **9（2.3）** | **3（5.9）** | **1（3.4）** | **10（4.0）** | **2.684** | **0.443** |
| **5-Minute Apgar Score (4-7points)** | **124（31.8）** | **27（52.9）** | **14（48.3）** | **129（51.2）** | **28.156** | **0.001** |
| **In Vitro Fertilization (IVF)** | **108（27.7）** | **12（23.5）** | **9（31.0）** | **58（23.0）** | **2.293** | **0.514** |
| ****Complications of extremely preterm infants**** |  |  |  |  |  |  |
| **Respiratory Distress Syndrome (RDS) Grade 3-4** | **94（24.1）** | **27（52.9）** | **19（65.6）** | **86（34.1）** | **21.538** | **0.001** |
| **Intraventricular Hemorrhage (IVH) Grade 3-4** | **49（12.6）** | **8（15.7）** | **8（27.6）** | **78（31.0）** | **34.239** | **0.001** |
| **Pulmonary Hemorrhage** | **20（5.1）** | **26（51.0）** | **8（27.6）** | **37（14.7）** | **94.872** | **0.001** |
| **Cerebral Softening** | **13（3.3）** | **0（0.0）** | **1（3.4）** | **11（3.5）** | **2.463** | **0.461** |

## ****Table 2.Univariate logistic regression analysis for extremely preterm infants mortality****

| **Variable** | **OR** | **CI lower** | **CI upper** | **P value** |
| --- | --- | --- | --- | --- |
| Gestational Age | 0.417 | 0.334 | 0.515 | 0.001 |
| Birth Weight | 0.996 | 0.995 | 0.997 | 0.001 |
| Female | 1.085 | 0.756 | 1.556 | 0.657 |
| Cesarean Section | 0.570 | 0.390 | 0.827 | 0.003 |
| Multiple Pregnancy | 1.838 | 1.272 | 2.664 | 0.001 |
| 1-Minute Apgar Score (0-3points) | 3.455 | 1.996 | 6.204 | 0.001 |
| 1-Minute Apgar Score (4-7points) | 1.193 | 0.840 | 1.697 | 0.325 |
| 5-Minute Apgar Score (0-3points) | 1.373 | 0.450 | 4.321 | 0.574 |
| 5-Minute Apgar Score (4-7points) | 2.461 | 1.717 | 3.545 | 0.001 |
| In Vitro Fertilization (IVF) | 0.857 | 0.564 | 1.297 | 0.468 |
| Gestational Hypertension | 1.239 | 0.773 | 1.988 | 0.373 |
| Gestational Diabetes Mellitus (GDM) | 0.708 | 0.421 | 1.172 | 0.184 |
| Premature Rupture of Membranes (PROM)≥18h | 0.790 | 0.550 | 1.133 | 0.202 |
| Abnormal Amniotic Fluid | 0.789 | 0.555 | 1.120 | 0.185 |
| Placental Abruption | 1.639 | 1.005 | 2.696 | 0.049 |
| Cervical Cerclage | 0.733 | 0.498 | 1.076 | 0.114 |
| Genital Tract Infection | 0.836 | 0.549 | 1.266 | 0.400 |
| Antenatal Magnesium Sulfate | 0.970 | 0.683 | 1.378 | 0.864 |
| Antenatal Corticosteroids | 0.411 | 0.250 | 0.664 | 0.001 |
| Antenatal Corticosteroids Full Course of Treatment | 0.430 | 0.292 | 0.626 | 0.001 |
| Respiratory Distress Syndrome (RDS) Grade 3-4 | 1.795 | 1.222 | 2.648 | 0.003 |
| Pulmonary Surfactant (PS) Administration | 0.149 | 0.050 | 0.364 | 0.001 |
| Multiple Doses of Pulmonary Surfactant (PS) | 2.306 | 1.581 | 3.382 | 0.001 |
| NO Administration | 10.625 | 3.003 | 67.481 | 0.002 |
| Vasoactive Drugs | 3.215 | 2.222 | 4.684 | 0.001 |
| Pulmonary Hemorrhage | 6.649 | 3.499 | 13.763 | 0.001 |
| Intraventricular Hemorrhage (IVH) Grade 3-4 | 2.509 | 1.597 | 3.998 | 0.001 |
| Cerebral Softening | 1.326 | 0.499 | 3.586 | 0.568 |

****Table 3 Multivariable logistic regression analysis of mortality in extremely preterm infants****

| **Variable** | **OR** | **CI lower** | **CI upper** | **P value** |
| --- | --- | --- | --- | --- |
| Gestational Age | 0.655 | 0.478 | 0.891 | 0.008 |
| Birth Weight | 0.999 | 0.997 | 1.000 | 0.168 |
| 1-Minute Apgar Score (0-3points) | 2.061 | 1.040 | 4.175 | 0.041 |
| 5-Minute Apgar Score (4-7points) | 1.337 | 0.842 | 2.116 | 0.216 |
| Antenatal Corticosteroids | 0.706 | 0.381 | 1.303 | 0.266 |
| Antenatal Corticosteroids Full Course of Treatment | 0.654 | 0.406 | 1.049 | 0.079 |
| Pulmonary Surfactant（PS）Administration | 0.113 | 0.035 | 0.309 | 0.001 |
| Multiple Doses of Pulmonary Surfactant（PS） | 1.604 | 0.973 | 2.641 | 0.063 |
| Vasoactive Drugs | 1.972 | 1.224 | 3.178 | 0.005 |
| Pulmonary Hemorrhage | 4.790 | 2.318 | 10.648 | 0.001 |
| Intraventricular Hemorrhage (IVH) Grade 3-4 | 1.366 | 0.782 | 2.387 | 0.272 |
| Multiple Pregnancy | 0.982 | 0.606 | 1.581 | 0.941 |
| NO Administration | 3.461 | 0.800 | 24.409 | 0.137 |
| Cesarean Section | 0.607 | 0.364 | 1.003 | 0.053 |
| Respiratory Distress Syndrome (RDS) Grade 3-4 | 1.421 | 0.861 | 2.344 | 0.168 |
| Placental Abruption | 1.324 | 0.716 | 2.446 | 0.369 |
